# Anthocyanin – Rich Red Dye of *Hibiscus Sabdariffa* Calyx Modulates Cisplatin-induced Nephrotoxicity and Oxidative Stress in Rats

**Published:** 2013-12

**Authors:** Adedayo O. Ademiluyi, Ganiyu Oboh, Oluwaseun J. Agbebi, Ayodele J. Akinyemi

**Affiliations:** 1 Functional Foods, Nutraceuticals and Phytomedicine Unit, Department of Biochemistry, Federal University of Technology, Akure, P.M.B. 704 Akure, 340001, Nigeria;; 2 Department of Biochemistry, Afe Babalola University, Ado – Ekiti, Nigeria, P.M.B. 5454, Nigeria

**Keywords:** Oxidative stress, cisplatin, nephrotoxicity, anthocyanin, *Hibiscus sabdariffa* calyx, red dye

## Abstract

This study sought to investigate the protective effect of dietary inclusion of *Hibiscus sabdariffa* calyx red dye on cisplatin-induced nephrotoxicity and antioxidant status in rats. Adult male rats were randomly divided into four groups of six animals each. Groups I and II were fed basal diet while groups III and IV were fed diets containing 0.5% and 1% of the dye respectively for 20 days prior to cisplatin administration. Nephrotoxicity was induced by a single dose intraperitoneal administration of cisplatin (7 mg/kg b.w) and the experiment was terminated 3 days after. The kidney and plasma were studied for nephrotoxicity and oxidative stress indices. Cisplatin administration caused a significant (*P*<0.05) increase in creatinine, uric acid, urea, and blood urea nitrogen (BUN) levels as well as kidney malondialdehyde (MDA) content, with concomitant decrease in kidney vitamin C and GSH contents. Furthermore, activities of kidney antioxidant enzymes such as, SOD, Catalase, and GST were significantly (*P*<0.05) altered in cisplatin administered rats. However, consumption of diets supplemented with the dye for 20 days prior to cisplatin administration protected the kidney and attenuates oxidative stress through modulation of *in vivo* antioxidant status. The determined anthocyanin content of the dye is 121.5 mg Cyanidin-3-rutinoside equivalent/100 g, thus, the observed nephroprotective effect of *H. sabdariffa* dye could be attributed to its anthocyanin content.

## INTRODUCTION

Cisplatin (*cis-*diamminedichloroplatinum (II), CDDP), an antineoplastic drug used in the treatment of many solid-tissue cancers has its chief side effect in nephrotoxicity (1). Nephrotoxicity involves kidney damage or dysfunction arising from direct or indirect exposure to drugs and industrial or environmental chemicals. Drugs such as cisplatin induce nephrotoxicity (2). The kidney which is the major route of cisplatin excretion also accumulates it to a greater degree than other organs (3, 4). Oxidative stress, inflammation, and apoptosis are some of the mechanisms already established to explain cisplatin-induced acute kidney injury (5). A number of strategies have been proposed for the prevention/management of cisplatin-induced nephrotoxicity since there is no specific treatment, with the use of some synthetic drugs been popular. However, these drugs have some associated risks and side-effects (6), hence the need for natural alternatives of plant origin (plant foods/extracts) with little or no side effect.

The use of plants with colour or dye for the prevention and management of diseases have been employed in folklore since time immemorial. Hibiscus (*Hibiscus sabdariffa* (L) Malvaceae) is one of the numerous species of hibiscus cultivated as an annual plant mainly for its flowers and fruit; used as spices, herbs and colourants (7). In South western Nigeria, dried red calyx of *Hibiscus sabdariffa* (English, Red Sorrel; Hausa, *Zobo*; Yoruba, Ishapa; Igbo, *Okworo-ozo*) and its extracts have been employed as infusion, colourant or dye in therapy for the management of arteriosclerosis and has also found use due to its diuretic, hypocholesterolemic, antihypertensive and mucolytic effects (8, 9).

The therapeutic roles of *Hibiscus sabdariffa* calyx and its extracts have been linked to its phytochemical constituents such as anthocyanin, phenolic compounds, flavonoids, protocatechueic acid; with anthocyanin being the most abundant due to the red colour of its extract (10). In light of recent findings, anthocyanin has been reported to posses vasoprotective and anti-inflammatory properties (11), inhibits lipid peroxidation and radical scavenging ability (12), anticancer and chemoprotective properties (13), as well as anti-neoplastic properties (14). Despite the known therapeutic properties of *Hibiscus sabdariffa* calyx extracts, there is dearth of information on its nephroprotective effect. Hence this study sought to investigate the protective effect of *Hibiscus sabdariffa* calyx dye on cisplatin-induced nephrotoxicity in rats.

## MATERIALS AND METHODS

### Materials

The calyces of *Hibiscus sabdariffa* flower were purchased at Erekesan market in Akure, Nigeria. Samples were authenticated at the Department of Crop, Soil and Pest management, Federal University of Technology, Akure, Nigeria. Calyces were air dried and pulverized prior to dye/pigment extraction. Cisplatin was sourced from Korea United Pharm. Inc. Bioassay kits were sourced from RANDOX Laboratories Ltd., Crumlin, Co. Antrim, UK. Except stated otherwise, all other chemicals and reagents were of analytical grade and the water was glass distilled. Diet ingredients were purchased from VITAL Feeds, Jos, Nigeria Ltd.

### Animals

The handling and use of the animals were in accordance with NIH Guide for the care and use of laboratory animals. Male albino rats weighing 165 ± 10 g were purchased from the animal colony, Department of Biochemistry, University of Ilorin, Nigeria. The animals were maintained at 25°C on a 12 hour light/dark cycle with access to food and water being ad libitum and prior to the commencement of the study, the animals were acclimatized under these conditions for two weeks. This study was approved by the Institutional Animal Ethical Committee of the Federal University of Technology, Akure, Nigeria.

### Extraction of *Hibiscus sabdariffa* calyx dye

The red dye was prepared according to Adetuyi *et al.* (15), but with slight modification. Briefly, the dye was extracted by soaking 100 g of the *Hibiscus sabdariffa* calyx powder in 1.8 L of distilled water and kept overnight (12 hours), and the mixture was filtered. The filtrate was collected and the residue was rinsed with another 200 mL of distilled water. This again was filtered and the filtrate collected and added to the previous one. The filtrate obtained was lyophilized and designated as the red dye used in this study.

### Quantification of total and monomeric anthocyanin in *Hibiscus sabdariffa* calyx dye

A modification of the pH differential method reported by Fuleki and Francis (16) was used for the quantitative determination of total and monomeric anthocyanin pigments. Briefly, 0.2 mL aliquots of the dye solution was diluted with 2.8 mL of buffer (consisting of 125 mL of 0.2 N KCl, and 385 mL of 0.2 N HCl), pH 1.0 and another 0.2 mL of the dye solution was diluted with 2.8 mL of buffer, (consisting of 400 mL of 1 N sodium acetate, 240 mL of 1 N HCl and 360 mL distilled water) solution pH 4.5. Thereafter, the absorbance of the two solutions was taken at 482 nm. Total anthocyanin pigments were determined using absorbance in pH 1.0 buffer, while monomeric anthocyanins were determined from the differences between absorbance in pH 1.0 and 4.5 buffers. And the anthocyanin content was calculated and expressed as mg Cyanidin-3-rutinoside equivalent /100 g of sample (Cyanidin-3-rutinoside, ε = 28840 M^-1^cm^-1^).

### Experimental design and induction of nephrotoxicity

The experimental animals were randomly divided into 4 groups of 6 animals each. Groups I and II were fed basal diet (50% skimmed milk, 36% corn starch, 10% groundnut oil and 4% mineral & vitamin premix), while Groups III and IV were fed basal diet supplemented with 0.5% and 1% of the dye respectively, for 20 days prior to cisplatin administration. The experimental diets were prepared according to the modified method of Oboh *et al*. (17). On day 20, Group I receive sterile water (1 mL/kg, i.p), while Group II, III and IV received a single dose intraperitoneal administration of cisplatin (7 mg/kg body weight) (18) and the experiment terminated 3 days after cisplatin administration. The animals were decapitated after an overnight-fast by cervical dislocation and the blood was rapidly collected by direct heart puncture into an EDTA bottle while the kidney was rapidly isolated, weighed and kept on ice.

### Analytical procedures

The plasma uric acid, urea, creatinine, and Blood Urea Nitrogen (BUN) were determined using commercially available kits (Randox Laboratories UK). Kidney lipid peroxidation was assayed using a slightly modified thiobarbituric acid (TBA) reaction (19) and quantified as Malondialdehyde (MDA) content. The activity of Superoxide dismutase (SOD) was determined as reported by Alia *et al*. (20), while catalase (CAT) and glutathione-*s*-transferase (GST) activities were quantified according to Sinha (21) and Habig *et al*. (22) respetively. Reduced glutathione (GSH) content was determined according to Ellman (23), and kidney vitamin C content was determined according to Benderitter *et al*. (24). The tissue total protein content was determined as reported by Lowry *et al*. (25).

### Data analysis

The results of replicate readings were pooled and expressed as mean ± standard deviation. One way analysis of variance was used to analyze the results and Duncan multiple range test was used for the post hoc (26). Statistical package for Social Science (SPSS) 16.0 for Windows was used for the analysis. The significance level was taken at *P<*0.05.

## RESULTS

The total and monomeric anthocyanin contents of the *Hibiscus sabdariffa* calyx red dye as revealed by this study are 121.5 mg Cyanidin-3-rutinoside equivalent/100 g and 102.7 mg Cyanidin-3-rutinoside equivalent/100 g) respectively.

The average feed intake of the rats per group is depicted in Table 1. The result revealed that the diets were well eaten by all the animals in various groups as indicated by their average feed intake during the period of the experiment. Furthermore, the effect of diets on the average weight gain/loss (%) of the treated animals revealed significant weight increase across the groups prior to cisplatin administration (unpublished data). However, there was a significant (*P*<0.05) weight loss in the cisplatin administered groups as compared with the normal rats, three (3) days after cisplatin administration. Nevertheless, this observed weight loss, was ably reversed in groups fed diets supplemented with *H. sabdariffa* calyx red dye prior to cisplatin administration (Table 1).

**Table 1:** Effect of diets supplemented with *Hibiscus sabdariffa* calyx dye on some kidney biochemical indices in cisplatin (7mg/kg i.p) administered rats

	Treatment Groups			
	I	II	III	IV

Average feed intake (g/rat/day)	9.2 ± 3.4^a^	9.1 ± 4.0^a^	9.0 ± 5.2^a^	9.5 ± 2.6^a^
Average weight gain/loss (%)	5.9^d^	-2.8^a^	-1.7^b^	-1.6^c^
***Kidneyvdamage markers (mg/dl)***				
Creatinine	1.1 ± 0.5^a^	2.2 ± 0.3^b^	1.9 ± 0.5^a^ ^b^	1.0 ± 0.9^a^
Uric acid	24.6 ± 18.9^a^	39.3 ± 9.7^a^ ^b^	45.1 ± 1.5^b^	63.9 ± 12.9^a^ ^b^
Urea	71.2 ± 11.4^a^	81.4 ± 4.6^a^	79.9 ± 1.5^a^	81.3 ± 0.8^a^
Blood Urea Nitrogen (BUN)	27.1 ± 5.3^a^	38.0 ± 2.2^c^	37.9 ± 0.7^b^	38.0 ± 0.4^c^
***Kidney enzymatic antioxidant indices***				
Catalase activity (mmol H_2_O_2_ consumed/min/mg protein)	0.6 ± 0.01^d^	0.3 ± 0.02^b^	0.2 ± 0.01^a^	0.4 ± 0.01^c^
GST activity (mmol CDNB conjugates formed/min/g protein)	22.2 ± 0.5^c^	11.0 ± 0.2^a^	11.4 ± 0.3^a^	15.3 ± 0.4^b^
SOD activity (Units/mg protein)	1.1 ± 0.02^c^	0.4 ± 0.01^a^	0.3 ± 0.02^a^	0.7 ± 0.02^b^
***Kidney non-enzymatic antioxidant indices***				
GSH (mg/100 g protein)	3.4 ± 0.1^c^	1.7 ± 0.4^a^	1.9 ± 0.4^a^	2.7 ± 0.7^a^ ^b^
Ascorbic acid content (mmol./100 g protein)	2.2 ± 0.3^c^	1.5 ± 0.4^a^	1.4 ± 0.1^b^	1.9 ± 0.9b^c^
MDA content (mmol./100g protein)	4.8 ± 1.4^a^	8.0 ± 1.3^b^	4.2 ± 0.7^a^	3.8 ± 1.2^a^

Values represent mean ± standard deviation (n=6).

a

b

c

d Values not sharing the same superscript letter on the same row are significantly (*P<*0.05) different. Groups I (normal rats fed basal diet), Group II (control rats administered cisplatin and fed basal diet), Group III (cisplatin administered rats fed diet supplemented with 0.5% *Hibiscus sabdariffa* calyx dye) and Group IV (cisplatin administered rats fed diet supplemented with 1% *Hibiscus sabdariffa* calyx dye).

Furthermore the modulatory effect of the *H. sabdariffa* calyx red dye supplemented diets on kidney function (Table 1) as exemplified by plasma creatinine, uric acid, urea and blood urea nitrogen (BUN) levels was also studied. And the finding revealed significant (*P*<0.05) elevation in plasma creatinine (2.2 mg/dL), uric acid (39.3 mgdL), urea (81.4 mg/dL) and blood urea nitrogen (BUN) (38.0 mg/dL) levels in the control rats compared with the normal rats, which was significantly (*P*<0.05) reduced to near normal in rats fed diets supplemented with *H. sabdariffa* calyx red dye (except plasma uric acid).

The modulatory effect of the *H. sabdariffa* calyx red dye supplemented diets on kidney non-enzymatic antioxidant indices such as, GSH, ascorbic acid and MDA contents revealed a significant (*P*<0.05) depletion/reduction in these kidney antioxidant indices. However, supplementation of the diets with *H. sabdariffa* calyx dye protected against alteration in the kidney non-enzymatic antioxidant indices in Group III and IV animals respectively (Table 1). In addition, the effect of the red dye diet supplementation on kidney enzymatic antioxidant indices such as superoxide dismutase (SOD), glutathione-*s-*transferase (GST) and catalase (CAT) activities was also studied (Table 1). This study revealed a significant reduction in the kidney activities of SOD, GST and catalase in cisplatin administered control group as compared with the Group I. However, supplementation of the diets with *H. sabdariffa* calyx red dye protect against depletion of these kidney antioxidant enzymes in rat groups fed diets supplemented with the red dye (Group III-IV).

## DISCUSSION

The inclusion of *Hibiscus sabdariffa* calyx dye in diets fed to rats in each group affected neither appetite nor causes loss of body weight. This finding is consistent with an earlier study on the effect of *H. sabdariffa* extract in diabetic rats (27). Furthermore, the weight loss observed after cisplatin administration is consistent with earlier study (28). However, this observed weight loss was not severe in rats fed diets supplemented with 0.5% and 1% *H. sabdariffa* calyx dye compared with the control group (Table 1); thus suggesting that *H. sabdariffa* calyx could protect against cisplatin – induced weight loss (27).

The drastic elevation in the kidney function biomarkers such as creatinine, uric acid, urea, and blood urea nitrogen (BUN) have been suggested to be indicative of imparied renal function (29, 30). Thus, observed elevation of these indices in cisplatin – administered rats is an indication of cisplatin – induced alteration in kidney function and hence nephrotoxicity; and this finding is consistent with those of earlier studies (31, 32). Hence, the observed reduction in the plasma creatinine and urea levels in the red dye treated groups suggests nephro – protective properties of *H. sabdariffa* calyx dye; and it is consistent with earlier report where *H. sabdariffa* calyx caused a reduction in creatinine levels and improved kidney functions (33). This properties, however, could be a function of some bioactive phytochemicals present in the *H. sabdariffa* calyx such as anthocyanins. Findings have shown that dried calyx of *H. sabdariffa* is rich in various phytochemicals such as anthocyannins, tannins, phenolic acids, phytosterols and policosanols, with potentials to significantly impact human health (34). Anthocyanins have been shown to posses several therapeutic properties such as, hepatoprotective and anti-inflammatory, anti-cancer and chemoprotective, as well as anti-neoplastic properties (34, 35). The increase in the kidney MDA content (Table 1) in the control rats suggests oxidative stress as a result of increased lipid peroxidation. This is consistent with earlier studies on cisplatin administration causing inflammation and lipid peroxidation (2, 3). And the increased kidney MDA content could be as a result of increased ROS production arising from the depletion of kidney antioxidant enzymes such as; SOD, GST and catalase (Table 1). Study has revealed that depletion in rat plasma SOD, catalase and GST resulted in increase MDA concentration due to lipid peroxidation (36). Thus, the observed alteration in the endogenous antioxidant status in the experimental animals suggests, cisplatin–induced nephrotoxicity could be as a result of oxidative stress or suppression of the antioxidant enzymes, as previously reported (36, 37). However, the reduction of the kidney MDA content and restoration of SOD, GST and catalase activities in red dye treated groups suggests an improvement in their endogenous antioxidant status, which could be due to the presence of anthocyannins and some other phytochemicals of physiological relevance in *H. sabdariffa* calyx red dye. Anthocyannins are potent antioxidants, capable of inhibiting lipid peroxidation and scavenging reactive oxygen species such as ^•^OH (38). Previous studies have demonstrated the antioxidant and drug detoxification potentials of *H. sabdariffa* calyx anthocyannins extracts (39, 40).

Cisplatin administration caused a drastic reduction in kidney GSH content in the control rats (Table 1). Reduced glutathione (GSH) plays a significant role as an antioxidant agent in animals. And could function as a scavenger of ROS such as, hydroxyl radicals, singlet oxygen (37) and could interact directly or indirectly with various oxidizing molecules, thus neutralizing them. Hence, the observed depletion in the kidney GSH content of the cisplatin administered rats could be due to the interaction of cisplatin with molecules contain sulphydryl groups such as GSH (41). However, restoration of GSH levels in groups treated with diets supplemented with *H. sabdariffa* calyx red dye suggests an improvement in their endogenous antioxidant status which could be a function of the anthocyanin content of the red dye. Anthocyanins belong to a group of plant phytochemical known as polyphenols. Polyphenols are strong antioxidants and could provide a sparing effect on the in vivo GSH store, mop-up free radicals and augment the body’s antioxidant status (10, 34).

Furthermore, the depletion in the kidney ascorbic acid content in the control rats may be related to depleted GSH storage because, GSH is also necessary for the recycling of ascorbic acid (42). Study has reported a significant decrease in plasma ascorbic acid levels following cisplatin administration in humans (43). Therefore, the observed improvement in the kidney ascorbic acid levels in the groups fed with diets supplemented with *H. sabdariffa* calyx red dye also suggests improvement in their antioxidant status. Ascorbic acid (vitamin C) is one of several reported exogenous antioxidants that helps build up the body’s defences against free radicals (39). Previous studies have demonstrated that *H. sabdariffa* calyx contains natural antioxidants like ascorbic acid which exhibits good nephroprotective properties (27, 39).

The presence of anthocyanins (monomeric and polymeric) at significant levels in the red dye further suggests that *H. sabdariffa* calyx dye could be used in therapy and management of nephrotoxicity due to it antioxidant properties and potential as natural food colourants (44). Anthocyanins have been employed as colourants in food preparation and confectionary production. In addition to impacting the desirable colour; anthocyanin has been reported to improve the antioxidant and therapeutic properties of foods.

In conclusion, cisplatin administration to rats induces nephrotoxicity and acute renal damage; which was suggested to be due to its depletion of their antioxidant status. However, this damage was ameliorated by diets containing *H. sabdariffa* calyx red dye, suggesting its possible antioxidant and therapeutic properties. Therefore, the use of the red dye of *H. sabdariffa* calyx as food colourants/additive could be a cheap dietary management strategy for the treatment of cisplatin toxicity and acute renal damage arising from the administration of this drug.
